# A Pilot Study on AI-Driven Age Estimation and Sex Determination in Greek Individuals

**DOI:** 10.3390/jimaging12060239

**Published:** 2026-05-29

**Authors:** Anastasia Mitsea, Nikolaos Christoloukas, Aliki Rontogianni, Marko Subašić, Denis Milošević, Marin Vodanović

**Affiliations:** 1 Department of Oral Diagnosis and Radiology, Dental School, National and Kapodistrian University of Athens (NKUA), 11527 Athens, Greece; 2 Division of Dental Technology, Department of Biomedical Sciences, University of West Attica, 12243 Athens, Greece; 3 Faculty of Electrical Engineering and Computing, University of Zagreb, 10000 Zagreb, Croatia; 4 Department of Dental Anthropology, School of Dental Medicine, University of Zagreb, 10000 Zagreb, Croatia

**Keywords:** AI, dental age estimation, sex determination, gender determination

## Abstract

AI methods (machine learning and deep learning methods) presented promising results concerning the accuracy of dental age estimation and sex determination. Therefore, this pilot study aims to evaluate the efficacy of an artificial intelligence system to estimate age and determine sex in a Greek population sample. Panoramic radiographs from 110 adult subjects comprised this study’s sample. Males and females were equally distributed (1/1) in the sample. The dental status of each patient was different. The sample’s age ranged from 9 to 84 years of age, with a mean age of 48.87 years (±16.14 yrs). The methodology employed beta versions of convolutional neural networks (CNNs) developed by the University of Zagreb. Separate CNNs were trained on 4000 panoramic radiographs: one for sex estimation and another for age estimation. The AI program overestimated the subjects’ age on average by 4.16 years. A statistically significant correlation was found between true and estimated sex (*p*-value < 0.001). In males, the rate of agreement was 56.36%, while for females it was 89.47% (z-test for two proportions; *p*-value < 0.001). For the overall sample, Kappa = 73.21%, indicating a very good agreement. The results concerning age estimation are not quite satisfactory and further research is needed.

## 1. Introduction

One of the core procedures in forensic dentistry is dental age estimation in living individuals or unidentified corpses. Even when identification is impossible, dental age estimation and sex determination are required to reconstruct the biological profile of the unidentified individual. Dental age estimation is crucial in medicolegal cases, asylum seeker cases, mass disasters and in archeological research [1,2,3,4,5].

Age estimation is either based on dental age or skeletal age. As it is evidence-based, dental age is more strongly correlated with chronological age than skeletal age. Numerous dental age estimation methods have been presented based on the radiographic evaluation of tooth development. After tooth development is complete, dental age estimation is based on the evaluation of age-related structural alterations in the teeth [2,3,6,7,8]. The different phenotypes, hormonal system, and patterns of body development and growth between the two sexes are the consequence of their different sex chromosomes. In forensic dentistry during sex determination procedures, we evaluate the significant differences between the two sexes that can be observed in the dentition and the craniofacial system [9,10,11,12,13].

Not only do dental age estimation methods differ in accuracy and reliability, but sex determination methods also vary in accuracy and reliability, as they are based on different population samples and employ different statistical approaches [2,3,9,10,11,12,13].

Although all the expert-based methods remain the standard of reference, they have several drawbacks. In addition to being subjective and time-consuming, they also require the involvement of a well-trained and experienced examiner. In complicated cases, when there is a disagreement between the experts or when the available evidence is inadequate, the impact of the above disadvantages may be crucial [6,7,9,10,11,12,13].

According to the current evidence, AI methods (machine learning and deep learning methods) have presented promising results concerning the accuracy of dental age estimation and sex determination. However, the performance of artificial intelligence models in different populations still presents some uncertainty related to the population samples, the quality of radiographic images, the acquisition protocols, the equipment used and the dentofacial anatomical variations [14,15,16,17,18].

These restrictions may be crucial in forensic cases, since they might cause legal implications. Consequently, specific population development and testing of AI models is necessary. Accordingly, before we apply these models in real cases concerning the Greek population, it is necessary to test how accurate these models are in the specific population. Therefore, this pilot study aims to evaluate the efficacy of an artificial intelligence system to estimate age and determine sex in a Greek population sample.

## 2. Materials and Methods

The ethical standards of the Declaration of Helsinki and guidelines of the Strengthening the Reporting of Observational Studies in Epidemiology (STROBE) statement were respected during the design and implementation of this study [19]. Ethical approval for this retrospective study was obtained from the Research and Ethics Committee of the School of Dentistry, National and Kapodistrian University of Athens, Greece. The panoramic radiographs we used were not acquired for the purpose of this study but rather for the patients’ treatment planning. Based on the study’s retrospective design, no informed consents were obtained from the participants.

Radiographs that belonged to that were artifact free completely edentulous patients or patients with bone pathologies, syndromes, or clefts were excluded. Only radiographs of good quality that were artifact-free were recruited. From each patient’s file, age, sex, and the date that the panoramic radiograph was performed were recorded. Each radiograph was anonymized before analysis processing.

Panoramic radiographs from 110 subjects comprised this study’s sample. Males and females were equally distributed (1/1) in the sample. The dental status of each patient was different. The sample’s age ranged from 9 to 84 years of age, with a mean age of 48.87 years (±16.14 yrs).

The methodology employed beta versions of convolutional neural networks (CNNs) developed by the University of Zagreb. Separate CNNs were trained on 4035 orthopantomograms of Croatian subjects: one for sex estimation [20] and another for age estimation [21]. In particular, this dataset consisted of 2368 orthopantomograms belonging to females and 1667 to males, with a mean age of 38.17 years, divided into 5-year age groups (Table 1). One of the authors uploaded the images to the experimental online application and recorded the estimated age and assessed sex.

## 3. Statistical Analysis

The distribution of true and estimated age and sex and the sex accuracy rate were investigated graphically. A paired *t*-test was applied to investigate the difference between true and estimated age for overall sample and by sex. Moreover, Lin’s rho concordance correlation coefficient of agreement between the true and estimated age was calculated, and Bland and Altman’s plots of agreement were produced, both for the overall sample and by sex. The distribution of accuracy (defined as the difference true age and estimated age) was investigated by evaluating the mean squared error (MSE) in 10-year age groups in the overall sample. The chi-square test was applied to assess the correlation between true and estimated sex distribution, while the Fleiss’ Kappa measure of agreement was used to investigate the rate of their agreement. The z-test for comparing two proportions was applied. Two-tailed *p*-values are reported. A *p*-value less than 0.05 was considered statistically significant.

## 4. Results

**Figure 1:**
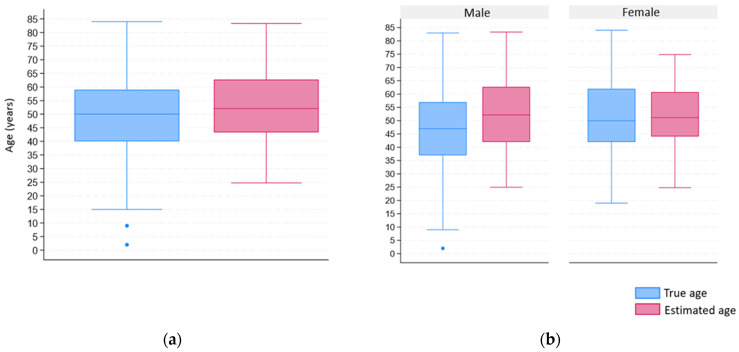
Box plots presenting the distribution of true and estimated age (years) (**a**) for overall sample and (**b**) by sex.

In the overall sample, a statistically significant mean difference was found between the true and estimated age (years): this was −4.16, with a 95% Confidence Interval of (−6.40 to −1.91) years. Therefore, the AI program overestimated the subjects’ age on average by 4.16 years (Table 2). In men, the AI program on average overestimated the male subjects’ age by 5.9 years, with a 95% Confidence Interval of (2.36 to 9.44) years. In women, no significant mean difference was observed between true and estimated age: the difference was −2.46 years, with a 95% Confidence Interval of (−5.29 to 0.37) years (Table 3, Figure 1).

**Figure 2:**
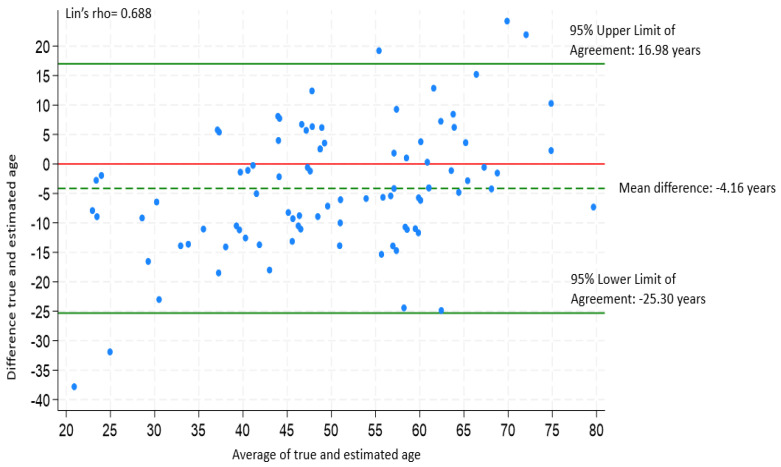
Scatter plot of difference vs. average true and estimated age (years) and 95% Limits of Agreement in the overall sample. The solid green lines represent the 95% Limits of Agreement, the green dashed line represents the mean difference between true and estimated age, and the red line represents a difference of zero.

**Figure 3:**
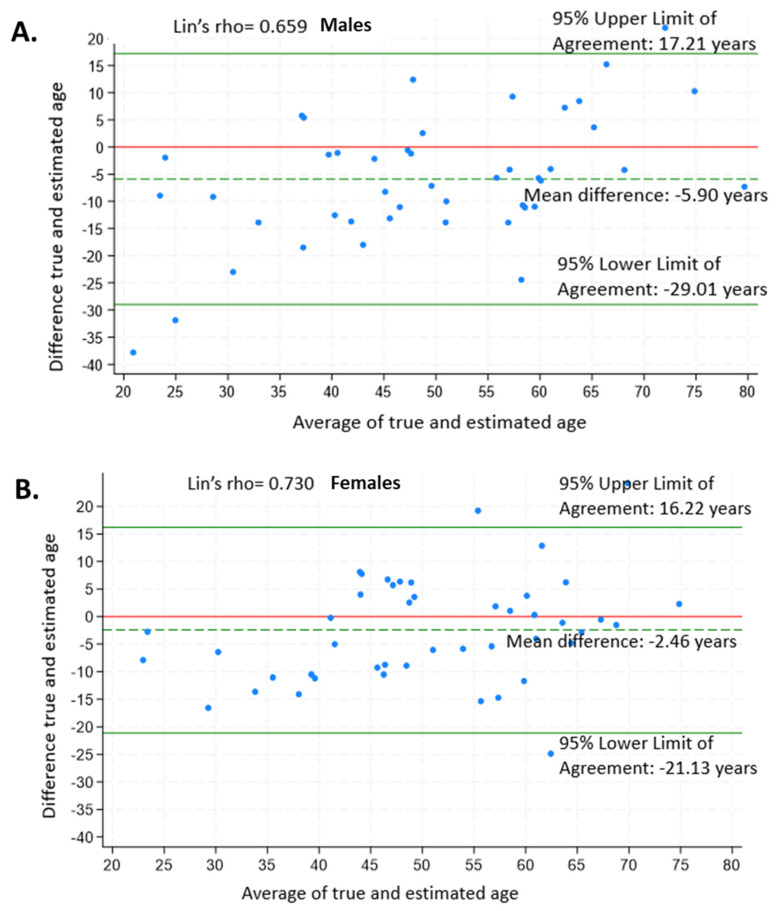
Scatter plots of difference vs. average true and estimated age (years) and 95% Limits of Agreement by sex ((**A**) males and (**B**) females). The solid green lines represent the 95% Limits of Agreement, the green dashed line represents the mean difference between true and estimated age, and the red line represents a difference of zero.

In the overall sample, Lin’s rho was 0.688, indicating a moderate agreement between true and estimated age. Moreover, the 95% Limits of Agreement are wide (−25.03 to 16.98) (Figure 2). When calculating Lin’s rho separately by sex, the agreement was higher in females compared to males (0.730 and 0.659, respectively), indicating a moderate agreement between true and estimated age. The 95% Limits of Agreement are wide: from −29.01 to 17.21 and −21.13 to 16.22 (Figure 3).

The 10-year age groups with the highest accuracy were 51–60 years and 61–70 years. On the other hand, the lowest accuracy in predicting age was observed in children (<10 years) and in the elderly (80+ years). However, this may be due to the small sample size for these age groups (Table 4).

**Figure 4:**
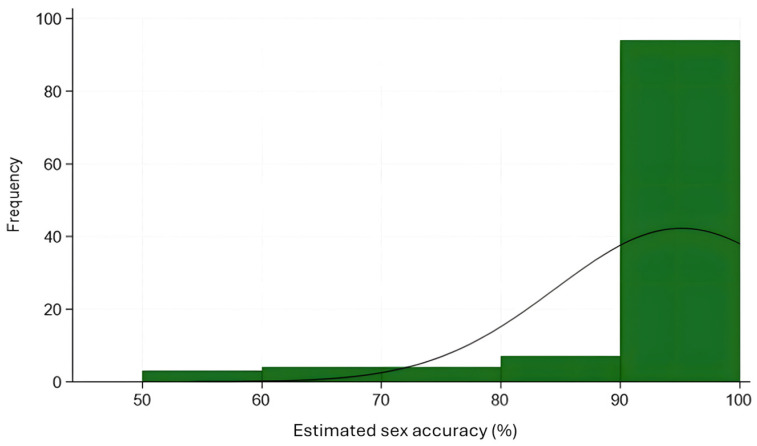
Histogram showing the distribution of estimated sex accuracy (%).

**Figure 5:**
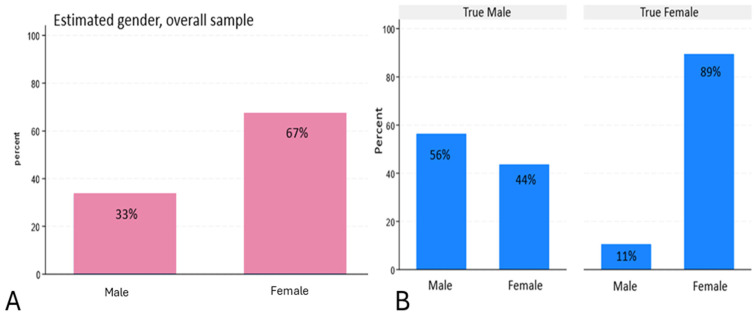
Bar plots presenting the distribution of (**A**) overall sample and (**B**) estimated sex by sex.

A statistically significant correlation was found between true and estimated sex (*p*-value < 0.001). In males, the rate of agreement was 56.36%, while for females the rate was significantly higher at 89.47% (z-test for two proportions; *p*-value < 0.001) (Table 5, Figure 4). The rate of agreement for the overall sample was Kappa = 73.21%, indicating a very good agreement beyond chance. However, the rate estimated by the Kappa agreement is significantly lower than the sex accuracy rate estimated by the AI program (73.21% vs. 95.12%, respectively; z-test for two proportions; *p*-value < 0.001) (Figure 5).

In only one case concerning sex determination, and in 21 instances regarding age estimation, the results could not be provided, possibly due to domain shift or other parameters related to the images. Regarding dental age estimation, the accuracy appeared to be 60%.

## 5. Discussion

The dental age estimation and sex determination methods applied so far present several disadvantages. Since dental age estimation methods rely on measurements or optical evaluation of tooth developmental stages, their accuracy and reliability depend mainly on the experience of the examiner. They are also related to the specific population group characteristics in which they have been initially applied, but also to the statistical models applied to analyze the data [2,3,6,7]. Sexual dimorphism in dental tissues is typically mild compared to skeletal elements. Consequently, the application of AI systems for sex determination based on dental evidence appears to be more complicated than determining sex based on skeletal features. AI-based sex determination might be affected by age, dental status, specific population characteristics, and the quality of the available evidence [11,12,13,14,18].

Therefore, the use of AI is particularly promising, since these models can identify and simultaneously study non-obvious multivariate quantitative or qualitative patterns related to tooth eruption, tooth development and calcification, dimensions of the pulp chamber, dental status, etc., and use them for age estimation and sex determination. So far, evidence-based AI systems were unable to completely replace traditional dental estimation and sex determination procedures. AI systems can serve as complementary tools to enhance accuracy and objectivity [15,16,17,18,22,23,24,25,26,27,28].

This pilot study attempts to investigate how reliable this AI application is for dental age estimation and sex determination based on the study of panoramic radiographs. This AI system overestimated the age in the overall sample. The correlation between chronological and dental age was moderate for the overall sample, while it was higher for the female subjects of the sample. In the overall sample, a higher accuracy was observed in the age groups of 51–60 and 61–70. With an accuracy of 60%, the results for age estimation were considered moderately satisfactory. It seems that the image quality, the number of teeth, the pulp changes, and the number and kind of dental treatments may affect the accuracy of dental age estimation of this AI system.

A statistically significant correlation was observed between true and predicted sex in the overall sample, while females presented a higher correlation than males. With an accuracy of 96.09%, the results for sex determination were considered particularly satisfactory. This specific AI system seems more successful at predicting sex than age. This might be related to the fact that this artificial intelligence system can successfully algorithmically exploit the biologically important information contained in dental radiographs. According to our knowledge, no other research has used AI systems to estimate age and determine sex in a sample of the Greek population; the findings of this study cannot be compared to previous studies on the Greek population.

Kim et al. [14] developed a deep learning model to estimate age and determine sex from 2067 panoramic radiographs of Korean subjects that were grouped according to age and sex. Human examiners, with different levels of experience, also studied the same sample, and the results were compared. The human observers correctly identified males and females 43.36% and 59.75% of the time, respectively. The AI model predicted sex with an accuracy of 90.25%, while the accuracy of the observers was lower (ranging from 40.3% to 63%). Moreover, the AI model presented a high sensitivity: 93.94% for males and 86.79% for females. This is in agreement with the results of our study, in which the accuracy of sex determination approached 96.09%. According to Kim et al.’s study’s results, more accurate age estimations were performed by the AI model. For subjects younger than 10 years old, the performance of both the AI system and human observers was equal. In the age group of 20-year-olds, the most accurate age estimation was performed by the AI system. In the older age groups, the performance of the AI system was reduced, and it was almost equal to that of human observers in the age group of 50 years of age, while in the 60+ age groups, the performance of the AI system dropped more [14].

Murray et al. [17] used 4003 panoramic radiographs (1809 belonged to males and 2149 to females) from subjects originating from the central–western region of Brazil aged 8 to 22.9 years to train a CNN model to estimate if a subject is younger or older than 18 years of age. The accuracy of age estimation was 88%, while in our study the accuracy was lower, and the better accuracy was observed in the 51–60 and 61–70 age groups [17].

In a study conducted by Hundur Hiyari et al. [29], sex determination and age estimation were performed by three new CNN systems trained by panoramic radiographs on a Bosnian population. The model that performed sex estimation presented an accuracy of 95.98%, higher than the results of the present study. On the contrary, the two CNN models that performed dental age estimation presented high accuracy (97.90% and 96.12%), while in our study the accuracy was significantly lower [18].

Kurniawan et al. [29], in a pilot study concerning an Indonesian population, developed a CNN model for dental age estimation trained with 801 panoramic radiographs of subjects from 5 to 15 years of age. A lower accuracy was observed in the 6.5-year age group, while a higher accuracy was observed in the 12.5-year age group. Recently, Oliveira et al. [30] developed a deep learning model for sex and age prediction, using panoramic radiographs of Brazilian subjects aged 5–15 years. In particular, 1320 radiographs were used as training material, 440 radiographs as validation, and 440 as test material. The developed model presented high performances in sex determination and age estimation. Concerning the age estimation of both studies, the relevant age groups (<10 years) presented the lowest accuracy in our study. Concerning sex determination, the results of the second study were better than the results of the present study [30].

It is necessary to identify our study’s limitations. The sample size is not particularly large or variable. Also, the training of the beta version of the convolutional neural network (CNN) software we used was based on a limited number of institution-specific panoramic images of Croatian subjects. Moreover, this beta version required no specific criteria for OPGs, like resolution, magnification, or exposure protocols, and imposed no restrictions on the ethnic background of the subjects, parameters that might be related to its performance.

An important factor in increasing the accuracy and reliability of these models is to use as much data volume as possible (i.e., panoramic radiographs) to train them. In particular, concerning panoramic radiographs, it would be beneficial to use images captured with different equipment and acquisition parameters [16,31]. So, this AI model needs further training using a larger number of panoramic radiographs from different population groups, with various anatomy and dental statuses, acquired with different panoramic equipment and different exposure protocols. Particularly, to test the accuracy and reliability of this AI model for the Greek population, we should test a bigger sample in the future.

The AI techniques for age estimation and sex determination have the potential to offer numerous benefits if they can be successfully applied to different population groups. AI methods will overcome the challenges associated with traditional methods that rely on expert experience and are time-consuming and non-standardized. Since AI is a constantly growing field, its main goal, which is to improve objectivity and eliminate human error, will succeed quickly.

## 6. Conclusions

This fully automated analysis of panoramic images required no specific criteria for images and imposed no restrictions on the ethnic background of the subjects. That may have had an impact on the results of this study. Concerning sex determination, the rate of agreement was better for females than for males. The results concerning age estimation are not quite satisfactory, and further research is needed. It seems that the image quality, the number of teeth, and the number and kind of dental treatments may affect the accuracy of this software. The current limitations of this software are that it was trained on orthopantomograms of the Croatian population and that all images used for training were made on only one type of X-ray device. For even better results in the training process, orthopantomograms from different devices and populations should be used.

## Figures and Tables

**Table 1:** AI training dataset sex and age distribution.

Age Group	18, 20	20, 25	25, 30	30, 35	35, 40	40, 45	45, 50	50, 55	55, 60	60, 65	65, 70	75, 80	80, 85	85, 90
Females	21	337	419	399	325	246	167	185	132	75	30	24	6	0
Males	8	221	264	234	247	188	137	118	98	79	35	24	11	1
Total	29	558	683	633	572	434	304	303	230	154	65	48	17	1

**Table 2:** Distribution of true and estimated age (years) and also the results comparing the mean difference in age in the overall sample.

Age (Years)	Mean	95% Confidence Interval	*p*-Value
True age	48.96	(44.56 to 51.35)	
Estimated age	52.11	(49.60 to 54.62)	
Difference	−4.16	(−6.40 to −1.91)	<0.001 *

* Statistically significant result.

**Table 3:** Distribution of true and estimated age (years) and also the results comparing the mean difference in age by sex.

Males: Age (Years)	Mean	95% Confidence Interval	*p*-Value
True age	46.42	(41.03 to 51.81)	
Estimated age	52.32	(48.59 to 56.05)	
Difference	−5.90	(−9.44 to −2.36)	0.002 *
**Females: Age (Years)**	**Mean**	**95% Confidence Interval**	***p*** **-value**
True age	49.46	(45.12 to 53.79)	
Estimated age	51.91	(48.40 to 55.43)	
Difference	−2.46	(−5.29 to 0.37)	0.087

* Statistically significant result.

**Table 4:** Mean squared error (MSE) as a measure of accuracy (lower MSE, higher accuracy) in 10-year age groups for the overall sample.

Age Group	MSE	No Subjects
<10	1222.91	6
11–20	167.87	14
21–30	113.80	14
31–40	105.09	14
41–50	88.84	14
51–60	45.77	14
61–70	45.49	14
71–80	65.94	14
80+	356.48	6

**Table 5:** Correlation between true and estimated sex.

True Sex	Estimated Sex		Chi-Square *p*-Value	Kappa
	Males	Females		
Estimated Sex	56.36%	43.64%		
Difference	10.53%	89.47%	<0.001 *	73.21%

* Statistically significant result.

## Data Availability

The data that support the findings of this study are available from the corresponding author upon reasonable request.
